# Genome-Wide Association of Proprotein Convertase Subtilisin/Kexin Type 9 Plasma Levels in the ELSA-Brasil Study

**DOI:** 10.3389/fgene.2021.728526

**Published:** 2021-09-29

**Authors:** Isabela Bensenor, Kallyandra Padilha, Isabella Ramos Lima, Raul Dias Santos, Gilles Lambert, Stéphane Ramin-Mangata, Marcio S Bittencourt, Alessandra C Goulart, Itamar S. Santos, Jose G Mill, Jose E Krieger, Paulo A. Lotufo, Alexandre C. Pereira

**Affiliations:** ^1^Center for Clinical and Epidemiologic Research, University of São Paulo, São Paulo, Brazil; ^2^Laboratory of Genetics and Molecular Cardiology, Heart Institute (InCor), University of São Paulo Medical School Hospital, São Paulo, Brazil; ^3^Lipid Clinic, Heart Institute (InCor), University of São Paulo Medical School Hospital, São Paulo, Brazil; ^4^Inserm UMR 1188 DéTROI, Université La Réunion, Sainte Clotilde, France; ^5^Department of Physiological Sciences, Federal University of Espírito Santo, Vitória, Brazil

**Keywords:** genetic variation, genome-wide association study, atherosclerosis, proprotein convertase subtilisin/kexin type 9, colocalization analysis

## Abstract

Pharmacological inhibition of PCSK9 (proprotein convertase subtilisin/kexin type 9) is an established therapeutic option to treat hypercholesterolemia, and plasma PCSK9 levels have been implicated in cardiovascular disease incidence. A number of genetic variants within the PCSK9 gene locus have been shown to modulate PCSK9 levels, but these only explain a very small percentage of the overall PCSK9 interindividual variation. Here we present data on the genetic association structure between PCSK9 levels and genom-wide genetic variation in a healthy sample from the general population. We performed a genome-wide association study of plasma PCSK9 levels in a sample of Brazilian individuals enrolled in the Estudo Longitudinal de Saude do Adulto cohort (*n*=810). Enrolled individuals were free from cardiovascular disease, diabetes and were not under lipid-lowering medication. Genome-wide genotyping was conducted using the Axiom_PMRA.r3 array, and imputation was performed using the TOPMED multi-ancestry sample panel as reference. Total PCSK9 plasma concentrations were determined using the Quantikine SPC900 ELISA kit. We observed two genome-wide significant loci and seven loci that reached the pre-defined value of *p* threshold of 1×10^−6^. Significant variants were near *KCNA5* and *KCNA1*, and *LINC00353*. Genetic variation at the *PCSK9* locus was able to explain approximately 4% of the overall interindividual variations in PCSK9 levels. Colocalization analysis using eQTL data suggested *RWDD3*, *ATXN7L1*, *KCNA1*, and *FAM177A1* to be potential mediators of some of the observed associations. Our results suggest that PCSK9 levels may be modulated by *trans* genetic variation outside of the *PCSK9* gene and this may have clinical implications. Understanding both environmental and genetic predictors of PCSK9 levels may help identify new targets for cardiovascular disease treatment and contribute to a better assessment of the benefits of long-term PCSK9 inhibition.

## Introduction

Proprotein convertase subtilisin/kexin type 9 (PCSK9) is a key modulator of LDL receptor (LDLR) degradation and, consequently, LDL-cholesterol (LDL-C) serum levels. Gain-of-function mutations in *PCSK9* have been shown to cause familial hypercholesterolemia and increased cardiovascular risk (Hopkins et al., 2015). On the other hand, loss-of-function variants have been shown to associate with low LDL-C levels and reduced cardiovascular risk (Kent et al., 2017). Furthermore, plasma PCSK9 has been independently associated with other components of the lipid profile (Leander et al., 2016; Brumpton et al., 2019). As a result, pharmacological inhibition of PCSK9 became mainstream as a lipid reduction strategy (Reyes-Soffer et al., 2017).

Understanding the factors that modulate interindividual variability of PCSK9 plasma levels is important for the better understanding of individual responses to treatment as well as the identification of new targets for cardiovascular disease treatment. The use of unbiased genetic approaches has the potential to contribute to increase our understanding of the two.

We conducted a genome-wide association study (GWAS) in healthy individuals from the general population aiming at the identification of genetic variation associated to plasma PCSK9 levels.

## Materials and Methods

### Study Population

The study sample belongs to the Estudo Longitudinal de Saude do Adulto, NCT02320461 (ELSA-Brasil). For the present analysis, we included 810 participants that have both PCSK9 plasma levels and genome-wide genotype information.

The ELSA-Brasil study design and cohort profile have been published elsewhere (Aquino et al., 2012). Briefly, ELSA-Brasil enrolled 15,105 civil servants living in six large Brazilian urban areas (Belo Horizonte, Porto Alegre, Rio de Janeiro, Salvador, Sao Paulo, and Vitoria), aged between 35 and 74years at baseline. Information on sociodemographic, clinical history, family history of diseases, lifestyle factors, mental health, cognitive status, and occupational exposure was assessed from August 2008 to December 2010. Anthopometric, laboratory and imaging measurements were also obtained. In addition to baseline measurements, samples of plasma and DNA were collected and stored for further analysis at −80°C (Pereira et al., 2013). All participants signed an informed consent before enrollment. The study was conducted in accordance with the Declaration of Helsinki and was approved by the Research Ethics Committees and by the National Research Ethics Committee (CONEP).

Participants enrolled in the Sao Paulo site (5,061 people in total) without diabetes (exclusion criteria: fasting plasma glucose-FPG>126mg/dl and/or 2-h post-load glucose >200mg/dl and/or history of treatment with oral anti-diabetic agents or insulin), without cardiovascular, renal or hepatic diseases (exclusion criteria: self-reported history of medical diagnosis of these pathologies), and who did not report prescription of lipid-lowering agents, were eligible for a PCSK9 ancillary and exploratory study. From the 1,751 randomly selected participants fulfilling the inclusion criteria for subsequent PCSK9 plasma concentration measurements (Ramin-Mangata et al., 2020), we used the 810 who had genome-wide genotype information for the present analysis.

### Biochemical Analyses

A 12-h fasting blood sample was drawn in the morning soon after arrival at the research clinic, following standardized procedures for sample collection and processing. A standardized 75g oral glucose tolerance test was performed in all participants without known diabetes utilizing an anhydrous glucose solution. For measurement of fasting and post-load glucose, we used the hexokinase method (ADVIA 1200, Siemens); for fasting and post-load insulin, an immunoenzymatic assay, and for HbA1c, high-pressure liquid chromatography. Total cholesterol (TC), high-density lipoprotein-cholesterol (HDL-C), and triglycerides (TG) were measured with enzymatic colorimetric assays (ADVIA Chemistry). LDL-C was calculated using the Friedewald equation. When TG were≥400mg/dl, LDL-C was measured directly with an enzymatic colorimetric assay (ADVIA Chemistry).

Total PCSK9 plasma concentrations were determined using the Quantikine SPC900 ELISA kit (R&D Systems, Lille, France; Ramin-Mangata et al., 2020). Briefly, plasma samples were diluted 1: 20 in the calibrator diluent onto ELISA plates and incubated for 2h on a plate shaker at 450rpm. Wells were rinsed with wash buffer using an automated Hydroflex TECAN microplate washer. The detection HRP-conjugated antibody was added to each well and plates were incubated for 2h at 450rpm. Wells were rinsed. The TMB substrate solution was added to each well and plates were further incubated in the dark for 30min at 450rpm. Reactions were stopped by the addition of 0.2N acid sulphuric solution. Absorbance was read at 450nm with reference at 540nm on an Infinite 200 pro TECAN plate-reader. The same experimenter (SR-M) performed the PCSK9 measurements on the same site and at the same time. The reported intra-assay precision coefficient of variation was 5.4%, and the minimum detectable dose of human PCSK9 ranged from 0.030–0.219ng/ml.

### SNP Genotyping and Imputation

Genomic DNA extraction has been previously described (Chor et al., 2019). ELSA-Brasil DNA samples were genotyped using Axiom_PMRA.r3 array (ThermoFisher) and genotypes annotated using the Axiom_PMRA.na35.annot.db provided at the ThermoFisher site. Genotype calling was performed using Affymetrix Power Tools. Initial VCF file containing 850,483 variants fulfilled all quality criteria.

Imputation was performed using the Haplotype Reference Consortium Michigan Imputation Server using the TOPMED reference haplotype panel as reference. After imputation data were exported in the standard PLINK format, downstream QC procedures and statistical analysis were conducted using the latest PLINK^1^ and R software packages,^2^ installed on a Linux-based computation resource. Imputation markers were kept if R2>0.3, and minor allele frequency (MAF)>0.01. A HWE value of *p* <1×10^−20^ was used to control for potential genotyping clustering problems. Genetic population structure was studied through PCA analysis after LD-pruning of associated markers (see also Statistical Analysis section). A total of 11,524,071 SNPs were used for genome-wide analysis, 11,289,274 for autosomal, and 234,797 for X-chromosomal analyses.

### Colocalization Analysis

For colocalization analysis, we defined a window spanning 500 Kb center at the most associated variant in all regions classified as having a suggestive association signal. Information on all variants within this region was used for colocalization testing. We used the LocusFocus^3^ analytical approach for colocalization testing. Briefly, all genes residing in each selected region with their expression quantitative trait loci (eQTL) summary statistics available in GTEx were sequentially tested for colocalization with the results obtained for PCSK9 association. As reference LD structure, we used 1,000 genomes 2012 European LD matrix (our sample has approximately 80% European ancestry). Colocalization was tested against all 48 tissues available in GTEx and the most significant signal was selected.

### Statistical Analysis

PCSK9 levels were log-transformed for all analyses. Baseline categorical parameters are presented using frequencies (proportions), and continuous parameters are presented using mean±SD. Before GWAS, we adjusted a linear model for log (PCSK9) adjusting for age. The residuals of this model were used for GWAS as a continuous variable. Confounding effects for age, sex, smoking and BMI were later tested for all genome-wide and suggestive GWA hits.

Genome-wide association analyses were conducted using plink. We conducted two analyses – one without any further adjustment and one adjusting for the first four principal components. The threshold for genome-wide significance was set to *p*<5×10^−8^. Associations with *p*<1×10^−6^ were considered as suggestive and presented as a list of top SNPs.

Due to the high level of admixture and complex genetic population structure present in the Brazilian population, we conducted two different sensitivity analyses taking into consideration self-referred race and a particular individual position in a PCA plot generated using the 2 first principal components. Briefly, for the self-referred race sensitivity analysis, association summary statistics were generated in each of three established subgroups: whites, blacks and browns (“pardos” in Portuguese). For the PCA-defined subgroup analysis, we used k-means clustering with *k*=3 and defined three different subgroups with higher European, African and Native-American ancestries. Meta-analysis used a fixed-effect model and was calculated using plink –meta-analysis routine.

Local association plots were created using LocusZoom (Pruim et al., 2010). Local linkage disequilibrium structure was determined using Haploview (Barrett, 2009).

Mediation analysis was conducted for selected loci. To select mark for a genetic risk score for plasma PCSK9 levels, we determined independently associated variants at the PCSK9 genomic locus (cis-pQTL) through fitting a multiple linear regression model using 20 nominally associated markers at this locus and a stepwise variable selection procedure. Genetic risk score was derived as the sum of weighted genotypes by their final regression coefficients.

## Results

### Relationship Between Cardiovascular Risk Factors and Plasma Pcsk9

Clinical and laboratory characteristics of the ELSA-Brasil sample used in the present analysis are summarized in Table 1. Plasma PCSK9 levels were associated with TC (*p*=0.0006), TG (*p*=0.003), and LDL-C (*p*=0.003; Table 1).

**Table 1:** Clinical and laboratory characteristics of studied subjects according to tertiles of plasma PCSK9 concentrations.

	1st	2nd	3rd	Overall
	(*n*=270)	(*n*=270)	(*n*=270)	(*n*=810)
**log(PCSK9; ng/ml)**				
Mean(SD)	5.4(±0.16)	5.7(±0.057)	6.0(±0.14)	5.7(±0.26)
**Sex**				
Male	123(46%)	129(48%)	124(46%)	376(46%)
Female	147(54%)	141(52%)	146(54%)	434(54%)
**Age(years)**				
Mean(SD)	50(±7.9)	50(±8.8)	52(±7.9)	51(±8.2)
**Race**				
Black	38(14%)	24(9%)	34(13%)	96(12%)
Mixed	54(20%)	63(23%)	61(23%)	178(22%)
White	159(59%)	163(60%)	158(59%)	480(59%)
Asian	12(4%)	15(6%)	12(4%)	39(5%)
Indigenous	3(1%)	2(1%)	4(1%)	9(1%)
Missing	4(1.5%)	3(1.1%)	1(0.4%)	8(1.0%)
**Smoking**				
Never smoker	147(54%)	162(60%)	133(49%)	442(55%)
Former smoker	65(24%)	74(27%)	94(35%)	233(29%)
Smoker	58(21%)	34(13%)	43(16%)	135(17%)
**BMI(kg/m^2^)**				
Mean(SD)	26(±4.6)	27(±4.8)	27(±4.9)	27(±4.8)
**Hypertension**				
No	210(78%)	201(74%)	197(73%)	608(75%)
Yes	60(22%)	69(26%)	73(27%)	202(25%)
**Glucose(mg/dl)**				
Mean(SD)	110(±6.5)	110(±6.3)	110(±6.7)	110(±6.5)
**HbA1c(%)**				
Mean(SD)	5.2(±0.46)	5.2(±0.47)	5.2(±0.46)	5.2(±0.46)
**Uric acid(mg/dl)**				
Mean(SD)	5.6(±1.4)	5.7(±1.5)	5.7(±1.6)	5.6(±1.5)
**Total cholesterol(mg/dl)**				
Mean(SD)	210(±37)	210(±38)	220(±38)	**220(±38)** ^***^
**Triglycerides(mg/dl)**				
Mean(SD)	120(±65)	130(±76)	140(±88)	130(±77)^**^
**HDL-C(mg/dl)**				
Mean(SD)	55(±14)	56(±14)	56(±13)	56(±14)
**LDL-C(mg/dl)**				
Mean(SD)	130(±31)	130(±32)	140(±33)	**130(±32)** ^**^

** *value of p 0.001–0.01*;

*** *p<0.001*.

### Genome-Wide Association Analysis of PCSK9 Plasma Levels

**Figure 1:**
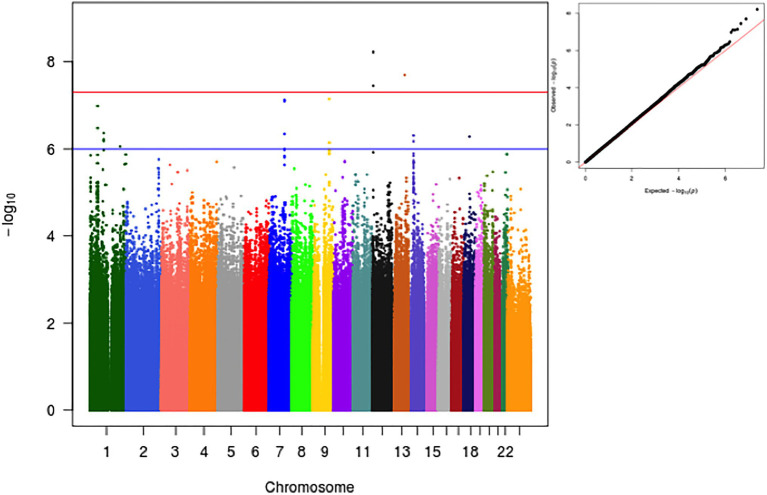
Manhattan and qqplot of GWA analysis for log-transformed PCSK9 as a function of variant, age, sex and the first 4 Principal Components.

We performed a GWA of the age-adjusted residuals of the log transformed values of plasma PCSK9. In the primary analysis adjusted for the four first principal components, we identified two loci that reached the pre-defined genome-wide significant level of 5×10^−8^ (Figure 1). Notably, no significant genomic inflation was observed (lambda=1.06). In addition, no significant difference was observed in the effect sizes and values of *p* in genome-wide significant and suggestive loci when running an unadjusted analysis (Supplementary Figure S1).

In addition, we explored the effect that genetic population structure might have on observed results. For this, we conducted two different sensitivity analyses. The first, involved a trans-ethnic meta-analysis after separating individuals into the three most commonly self-referred races in Brazil (i.e., Whites, Blacks and Browns; Supplementary Figure S2). In the second, we first derived clusters using a k-means cluster algorithm with *k*=3 and data from the 2 first PCs (Supplementary Figure S3). Samples were divided into 3 subgroups and analysis proceeded as described for the sensitivity analysis using self-referred race. Of note, the overall results were very similar to the overall analysis.

### Genome-Wide Significant Loci

In our main analysis, we observed two genome-wide significant loci and seven loci that reached the pre-defined value of *p* threshold of 1×10^−6^ (Table 2).

**Table 2:** Genome-wide and suggestive associated loci.

Marker	Chromosome	Position (Hg19)	Ref allele	Alt allele	value of *p*
rs35120342	1	52,497,077	G	T	1.03E-07
rs12125618	1	95,781,754	T	G	4.28E-07
rs17015194	1	206,911,203	C	G	8.80E-07
rs112386665	7	105,335,698	C	A	7.77E-08
rs4574919	9	113,501,372	T	G	7.18E-08
rs116367042	12	5,109,095	T	C	5.97E-09
rs9555910	13	90,188,022	T	G	2.00E-08
rs10444669	14	35,659,171	T	A	4.91E-07
rs12457651	18	34,790,416	T	C	5.24E-07

**Figure 2:**
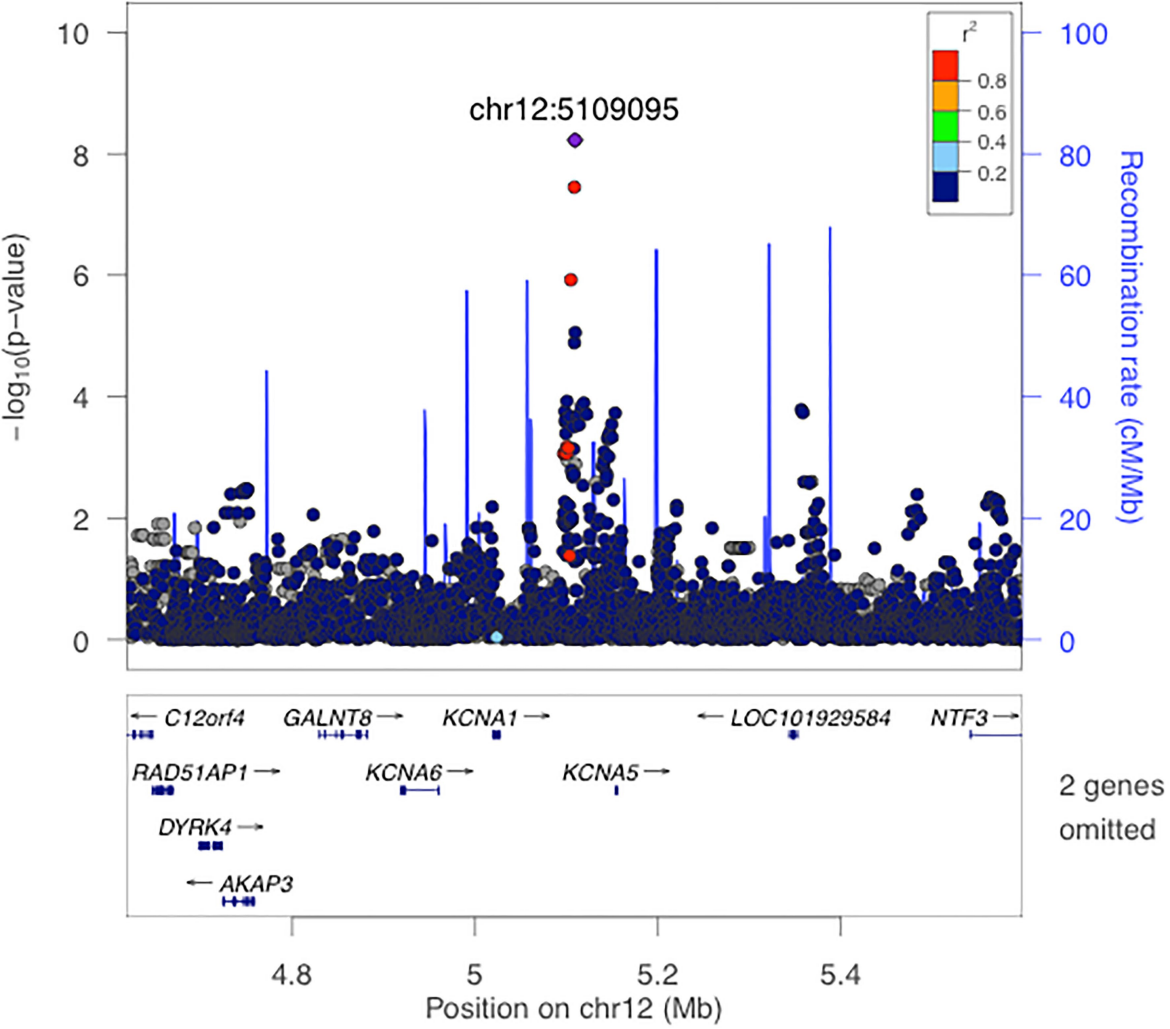
Local association plot for rs116367042.

The strongest associations with PCSK9 plasma levels were observed on chromosome 12p13.32, top lead SNP rs116367042 (value of *p* 5.97e-09). A regional association plot of the locus is shown in Figure 2. The closest gene *is KCNA5* and significant eQTLs have been observed in the region for *AKAP3*, *DYRK4*, *KCNA5*, *KCNA1*, *NDUFA9*, and *GALTN8*. The region has been described as associated with serum uric acid levels in a previous GWAS. All summary statistics from the main analysis can be found at Supplementary File 1.

The second genome-wide significant hit was observed in 13q31.2, in the region coding for *LINC00353*. A regional association plot of the locus is shown in Supplementary Figure S5. Notably, only a single marker was associated with PCSK9 levels at this locus, reducing, thus, its credibility.

### Suggestive Loci

Using a pre-defined suggestive significance threshold of 1×10^−6^ we identified additional 7 loci (Supplementary Table S2; Supplementary Figure S5). Those loci were on chr1p32.3 (nearest gene *TXNDC12*.), chr1p21.3 (nearest gene *RWDD3*), chr1q32.1 (nearest gene *MAPKAPK2*), chr7q22.3 (nearest gene *ATXN7L1*), chr9q31.3 (nearest gene *MUSK*), chr14q13.2 (nearest gene *KIAA0391*), and chr18q12.2 (nearest gene *KIAA1328*).

### PCSK9 Locus Association Structure

Previous GWAS and candidate-gene association studies have observed significant associations between PCSK9, LDL-C, and TC levels and genetic variants at the PCSK9 locus. Here we extend these observations using a multi-ethnic sample (Supplementary Figure S6). Of note, stronger associations are located at the 3′ region of PCSK9 and within the nearby *USP24* gene. Interestingly, previous studies in Europeans, African and other admixed samples have also described stronger associations for total cholesterol and LDL levels at this same region.

Linkage disequilibrium of the PCSK9 locus was resolved in four main haplotype blocks (Supplementary Table S1). Fifty-seven markers were nominally associated with PCSK9 levels being the most associated rs505151, rs662145, rs487230, and rs555687. Tagging associated SNPs in the PCSK9 locus, we were able to reduce the number of associated variants from 57 to 20, capturing 100% of the initial variation.

Using information from all 20 tagged markers and a stepwise regression approach, we were able to derive a PCSK9 instrumental variable made of four independently associated markers at the PCSK9 locus (cis-pQTLs; Supplementary Table S1). The R-squared for the multiple regression model containing all 4 markers was 0.036. Of particular importance, a model containing independently associated markers, BMI, age and smoking status, although highly significant (*p*=5.186e-08) was only able to explain 5.2% of the overall variation in PCSK9 plasma levels in our sample, genetic information being the variable with the highest effect size in our model.

### Colocalization Analysis of Associated Loci

Finally, we studied the colocalization pattern between the identified loci and expression traits of the genes in the vicinity of the association signal. For this, we used data from all the available tissues in the GTEx database. Colocalization analysis suggested that *RWDD3*, *ATXN7L1*, *KCNA1*, and *FAM177A1* are potential mediators of the observed associations on chromosomes 1, 7, 12, and 14, respectively (Supplementary Table S2).

## Discussion

PCSK9 is a serine protease with protein–protein interaction with the LDL receptor that has both genetic and clinical validation (Petrilli et al., 2020). PCSK9 binds to the LDLR and is thought to reduce the recycling of these proteins from the cell surface (sending them to lysosomes instead), inhibiting LDL-particle removal from the extracellular fluid (Deng et al., 2019). Blocking PCSK9 can lower blood LDL-C concentrations, and low PCSK9 levels are associated with lower LDL-C levels and reduced incidence of atherosclerotic cardiovascular disease. Despite the elusive importance of PCSK9 in lipoprotein homeostasis, few studies have analyzed PCSK9 plasma levels as a function of global genetic variation (Paquette and Baass, 2018; Pott et al., 2018). Understanding the genetic architecture that modulates PCSK9 levels may help dissect the mechanisms by which PCSK9 inhibition improves vascular function and overall cardiovascular morbidity and mortality.

It is assumed that PCSK9 modulates cardiovascular risk through cholesterol levels, more specifically LDL-C levels. Indeed, pharmacological inhibition of PCSK9 leads to significant decreases in LDL-C and reduction in the incidence of cardiovascular events. However, it is not known whether PCSK9 has other actions independent of plasmatic LDL-C levels (Cesaro et al., 2020), and the association between PCSK9 inhibition and inflammatory markers has not been as consistent as its association to lipid levels (Ruscica et al., 2019). For instance, PCSK9 is substantially expressed in arterial walls and macrophages (Cariou et al., 2016), and it is possible that its actions in these cells are not directly linked to LDL-C metabolism. PCSK9 has also been shown to be associated with metabolic factors other than lipoproteins. It is positively associated with albumin, liver enzymes (ALT, ALP, AST, GGT) and with hepatic steatosis, although whether this association is confounded by or mediated by LDL-C is still unclear (Paquette et al., 2020). Finally, PCSK9 is also expressed in the intestine, endocrine pancreas and brain, and non-lipid-lowering effects of PCSK9 inhibition could also be linked to platelet activation (Qi et al., 2021), cell proliferation and apoptosis (Macchi et al., 2021).

Importantly, there is great interindividual variation in both PCSK9 levels and response to PCSK9 inhibitors (Chasman et al., 2012; Qamar et al., 2019; Ramin-Mangata et al., 2020). In addition, only about 20% of circulating PCSK9 variance can be explained by clinical variables. Previously identified genetic variation only adds less than 5% to this figure, almost all of it from eQTL and pQTL within the PCSK9 locus itself (Anderson et al., 2014; Lim, 2017).

We conducted a GWAS study aiming at identifying genetic determinants of PCSK9 plasma levels. To our knowledge, this is the second GWAS conducted for PCSK9 levels and the first using a sample from a multi-ethnic population. Despite the relatively small sample size, we were able to observe two genome-wide significant association loci and a number of loci with suggestive association signals. In addition, we have confirmed the previously described association between PCSK9 levels and common genetic variation at the *PCSK9* locus (Pott et al., 2018). The most interesting observed genome-wide significant locus was at Chr12 within the *KCNA* gene cluster (Figure 2). Colocalization analysis was able to detect a significant colocalization signal with the *KCNA1* gene expression profile in adipose subcutaneous tissue. Mutations in *KCNA1* have been shown to cause episodic ataxia/myokymia syndrome type 1. The gene is lowly expressed in the adipose tissue and liver; and no metabolic phenotype has been associated with manipulations in *KCNA1*. Despite not being able to replicate this finding in an independent GWAS, further work characterizing the role of genetic variants nearby *KCNA1* in PCSK9 levels is warranted.

We are not the first to describe genome-wide significant variants associated with plasma PCSK9 levels outside of the *PCSK9* gene. Pott et al. in a GWAS conducted in 3290 individuals from the LIFE-Heart cohort identified variations within the *FBXL18* gene to be associated with PCSK9 levels (Pott et al., 2018). We did not identify any association in this region and together with the low imputation quality the authors of this previous GWAS described, we suggest the association between *FBXL18* and PCSK9 to be targeted in future studies aiming at clarifying the role of this locus on potentially regulating PCSK9 serum levels.

Plasma PCSK9 levels have been associated with several cardiovascular and metabolic risk factors (Caselli et al., 2019). Notwithstanding the understanding of PCSK9 mechanism at the molecular level, completely understanding the directionality of the associations between PCSK9 levels and other metabolic traits has been ill explored. In fact, most studies assume that PCSK9 is associated with lipid levels because of the interaction between PCSK9 and LDLR at the molecular level. However, it is unknown whether different predictors of PCSK9 levels are indeed associated with the same degree of increased cardiovascular risk. Indeed, recent experimental and clinical studies have also reported that higher circulating PCSK9 levels contributed to coronary atherosclerosis by enhancing the expression of pro-inflammatory genes, promoting apoptosis of human endothelial cells and activating platelet reactivity (Ricci et al., 2018; Yurtseven et al., 2020). The causal directionality of these associations, however, has not been fully explored and neither has the relationship of predictors of PCSK9 interindividual variation and clinically actionable management strategies.

By identifying potentially *trans* associations with PCSK9 levels, our data give rise to the possibility of a more complex mechanism, where different genetic factors may modulate *PCSK9*. It remains to be determined if PCSK9 levels driven by *trans* genetic factors carry the same increased risk of cardiovascular disease as PCSK9 levels determined by genetic variation at the *PCSK9* locus. It is important to note that similar *trans* mechanisms have been identified for other important genes regulating lipid metabolism, such as LPA (Li et al., 2015). In summary, our data suggest that PCSK9 levels may be modulated by upstream targets other than genetic variation in the *PCSK9* gene, which are well-known proxies for PCSK9 levels. The lack of large-effect size loci that modulate PCSK9 serum levels also points to the possibility that interindividual variation in *PCSK9* is mostly a function of epigenetic modulation and not of a polygenic component. Indeed, several studies have shown that the *PCSK9* promoter is dynamically methylated following several different exposures.

This study has some potential limitations. First and foremost, we have not been able to find a suitable replication sample for our GWAS results. The observed genome-wide significant loci still need to be replicated in an independent sample to be, in effect, taken as drivers of PCSK9 plasma levels (Trinder and Brunham, 2021). In addition, the reduced sample size of our study may have prevented us to identify other genome-wide significant loci with decreased effect size. In fact, post hoc calculation of our statistical power assuming a MAF of 0.2 was 0.73 to detect a difference of 0.5 standard deviation in the mean values of genotype groups, but only 0.11 to detect the same, different for alleles at a MAF of 0.1. Mendelian randomization analysis using our sample lacked the necessary statistical power to derive robust conclusions regarding the causality of *trans* PCSK9 variants and coronary artery disease or even LDL-C levels. Finally, lack of data on hsCRP and lipoprotein (a) precluded efforts in trying to understand the relevance of the described genetic associations in these variables. These aspects should be better defined in further studies.

## Conclusion

In conclusion, we describe new genome-wide significant loci associated with PCSK9 plasma levels in a sample from a healthy population. Our results suggest that PCSK9 levels may be modulated by *trans* genetic variation outside of the *PCSK9* gene. Understanding both environmental and genetic predictors of PCSK9 levels may help identify new targets for cardiovascular disease treatment and contribute to better assessment of the benefits of long-term PCSK9 inhibition.

## Data Availability Statement

All summary statistics from the main analysis are publicly available as Supplementary Material to this manuscript (Supplementary File Data Sheet 1). The data that support secondary findings of this study are available from ELSA-Brasil study on reasonable request. Requests for access to more detailed summary statistics, replication results, and analytic methods will be considered by the authors.

## Ethics Statement

The studies involving human participants were reviewed and approved by Research Ethics Committees and by the National Research Ethics Committee (CONEP). The patients/participants provided their written informed consent to participate in this study.

## Author Contributions

IB conceptualized and designed the study, researched data, and wrote the manuscript. KP, IL, RS, GL, MB, AG, IS, JM, and JK acquired data, assisted with the data analysis, and edited the manuscript. SR-M performed experiments and analysed and interpreted the results. PL and AP supervised the work, performed statistical analyses, and wrote the manuscript. All authors reviewed the manuscript.

## Funding

RS is recipient of a scholarship from Conselho Nacional de Pesquisa e Desenvolvimento Tecnológico, Brazil (CNPq) #303734/2018-3. MB is recipient of a scholarship from Conselho Nacional de Pesquisa e Desenvolvimento Tecnológico, Brazil (CNPq) #310255/2018-0. The funders had no role in study design, data collection and analysis, decision to publish, or preparation of the manuscript. The ELSA-Brasil baseline study was supported by the Brazilian Ministry of Health (Science and Technology Department) and the Brazilian Ministry of Science and Technology (Financiadora de Estudos e Projetos and CNPq National Research Council).

## Conflict of Interest

RS has received honoraria related to consulting, research and/or speaker activities from: Abbott, Ache, Amgen, Astra Zeneca, Esperion, EMS, Kowa, Libbs, Novo-Nordisk, Merck, MSD, Pfizer, PTC pharmaceuticals and Sanofi/Regeneron. MB has received honoraria related to consulting, research and/or speaker activities from: Boston Scientific, EMS, GE HealthCare, Novo-Nordisk, and Sanofi/Regeneron.

The remaining authors declare that the research was conducted in the absence of any commercial or financial relationships that could be construed as a potential conflict of interest.

The reviewer MR declared a past co-authorship with one of the authors RS to the handling editor.

## Publisher’s Note

All claims expressed in this article are solely those of the authors and do not necessarily represent those of their affiliated organizations, or those of the publisher, the editors and the reviewers. Any product that may be evaluated in this article, or claim that may be made by its manufacturer, is not guaranteed or endorsed by the publisher.

## Footnotes

^1^ http://pngu.mgh.harvard.edu/_purcell/plink

^2^ http://www.r-project.org/

^3^ https://locusfocus.research.sickkids.ca/
