# Supplementary material for: Genome-Wide Association of Proprotein Convertase Subtilisin/Kexin Type 9 Plasma Levels in the ELSA-Brasil Study
Source: Front Genet. 2021 Sep 29;12:728526. doi: 10.3389/fgene.2021.728526 (PMC8514075; doi:10.3389/fgene.2021.728526)
Supplement: Supplementary file 2 [file Data_Sheet_1.docx]

1. **Supplementary Figures**


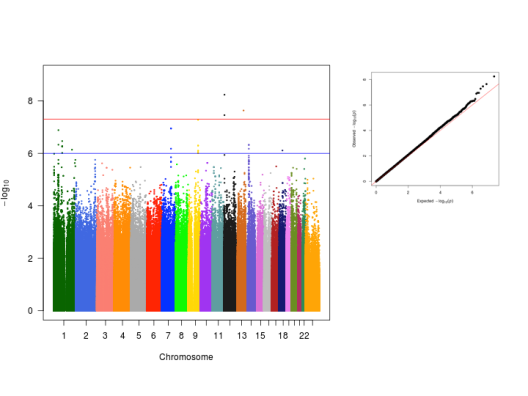


**Supplementary Figure** **S1.** Log-PCSK9 GWA adjusted for age.

**
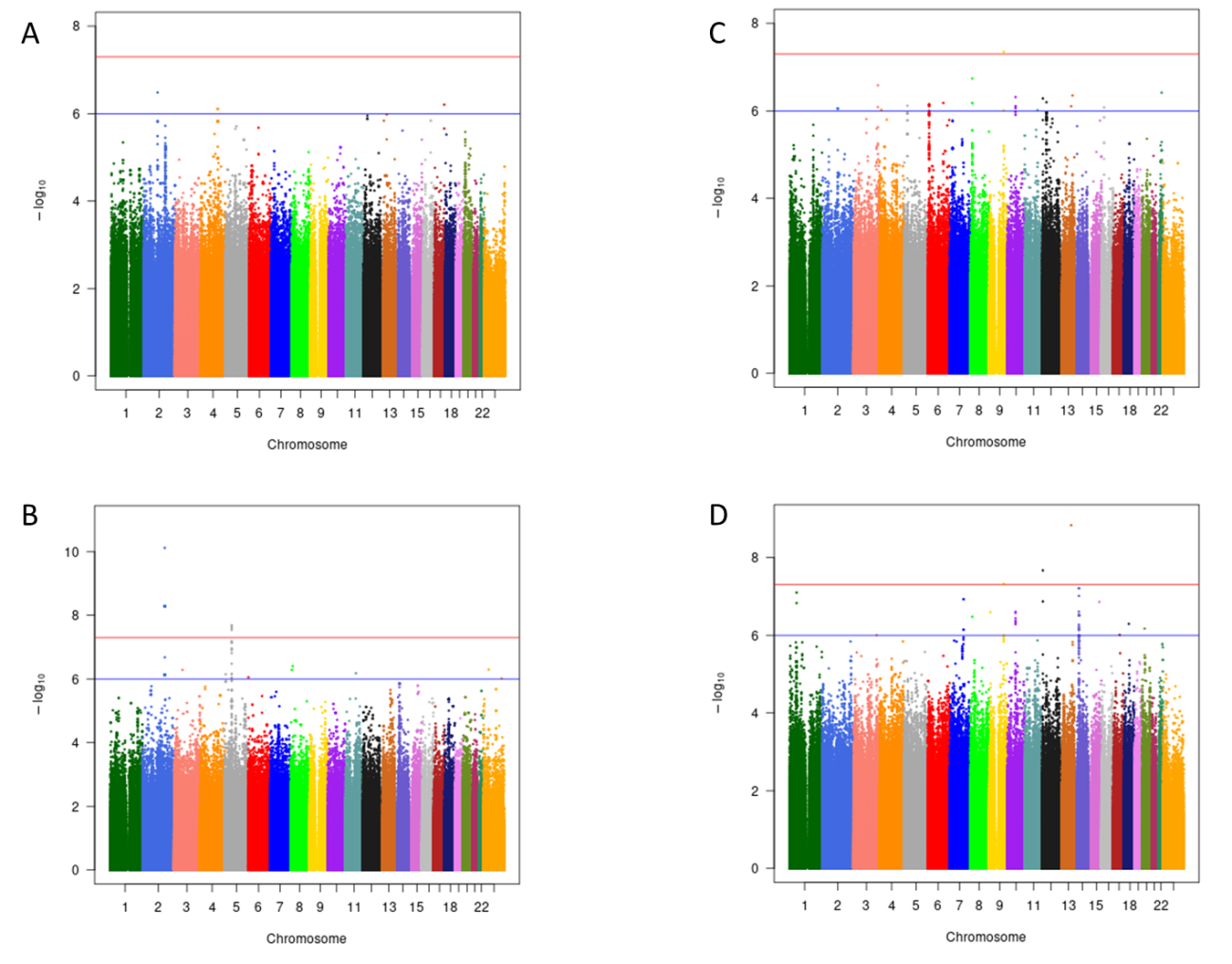
**

**Supplementary Figure S2**. Trans-ethnic meta-analysis using self-referred race to stratify groups. A. Self-referred Black; B. Self-referred Brown; C. Self-referred White; D. Fixed-effect meta-analysis overall result.


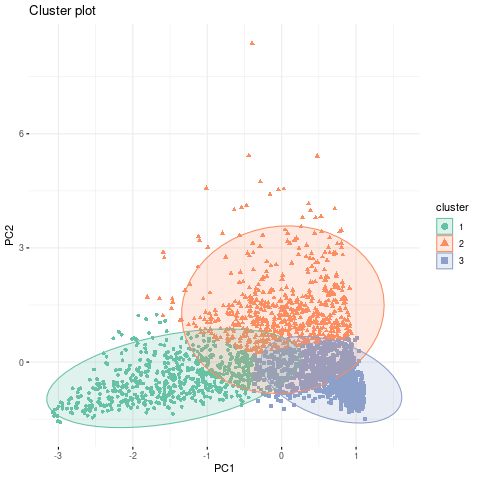


**Supplementary Figure S3.** PCA representation of studied samples. Clusters were defined using k-means clustering with k=3.


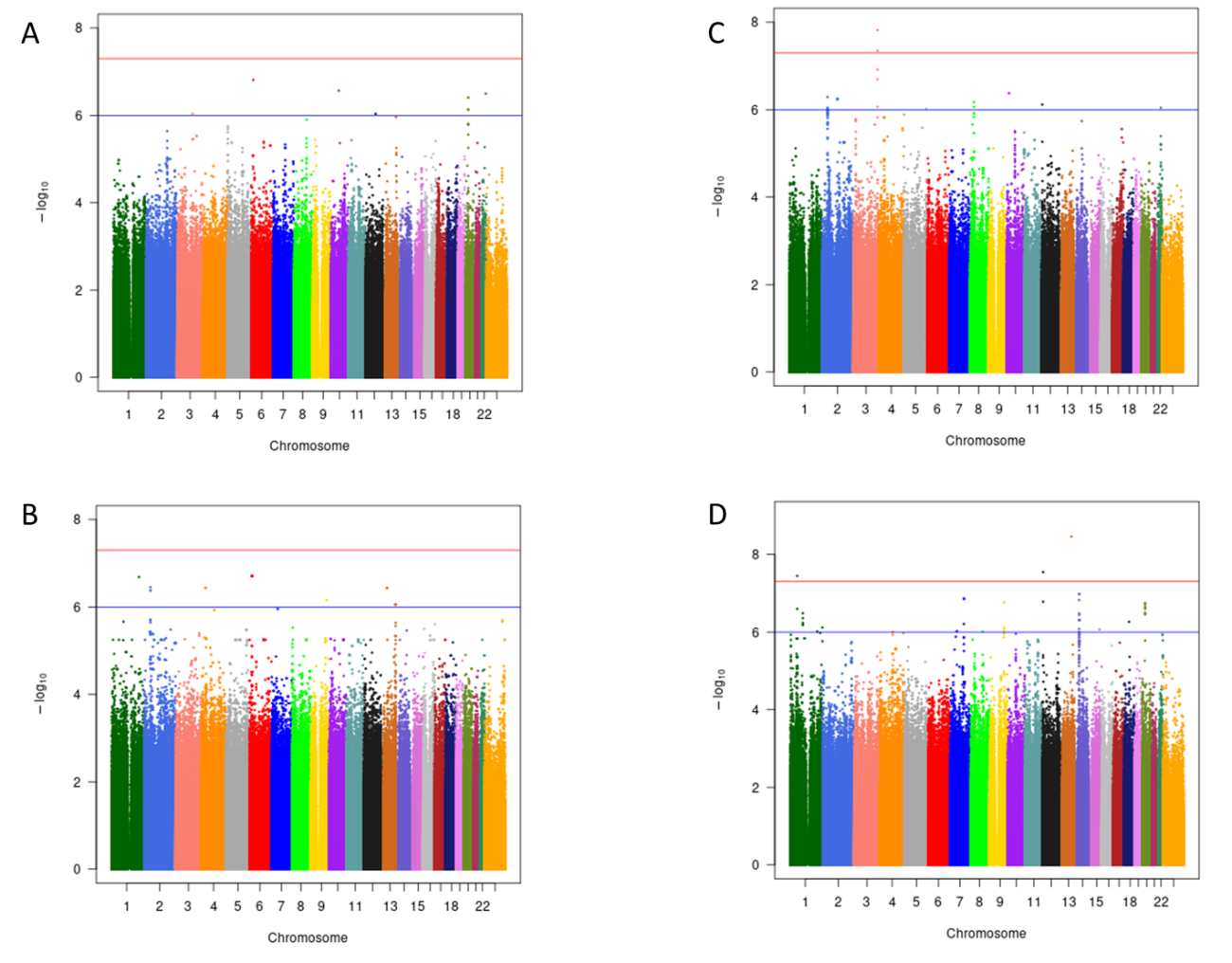


**Supplementary Figure S4.** Trans-ethnic meta-analysis using pca-defined cluster to stratify groups. A. African Cluster; B. Native-American Cluster; C. European Cluster; D. Fixed-effect meta-analysis overall result.


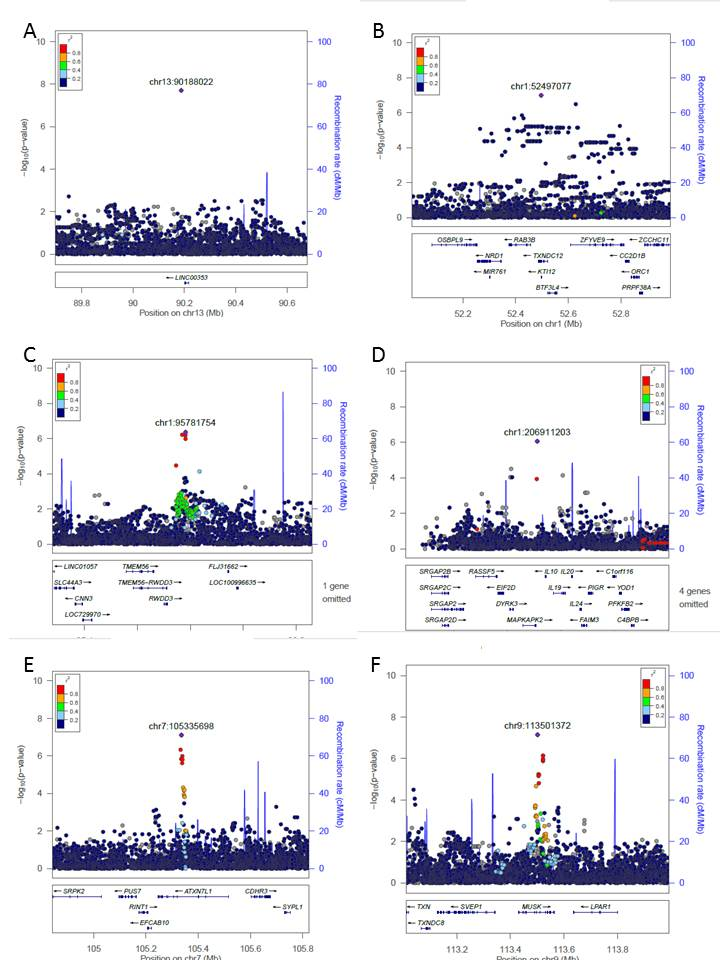


**Supplementary Figure S5**. Suggestive associated loci. Those loci were on 13q31.2, in the region coding for *LINC00353* (A); chr1p32.3 (nearest gene *TXNDC12*, B), chr1p21.3 (nearest gene *RWDD3*, C), chr1q32.1 (nearest gene *MAPKAPK2*, D), chr7q22.3 (nearest gene *ATXN7L1*, E) , chr9q31.3 (nearest gene *MUSK*, F), chr14q13.2 (nearest gene *KIAA0391*, G), and chr18q12.2 (nearest gene *KIAA1328*, H).


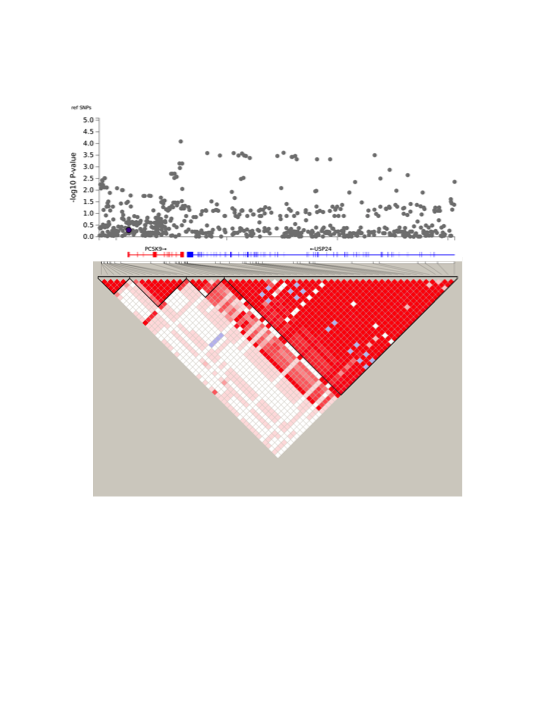


**Supplementary Figure S6.** Local association structure at the PCSK9 gene locus (upper panel); local linkage-disequilibrium structure at the PCSK9 gene locus.

1. **Supplementary tables**

| **Supplementary Table 1.** Multiple linear regression for PCSK9 instrumental variable | | | | | |
| --- | --- | --- | --- | --- | --- |
|  |  |  |  |  |  |
|  | Estimate | Std. Error | t value | Pr(>\|t\|) |  |
| (Intercept) | 0.07977 | 0.094 | 0.849 | 0.396358 |  |
| chr1:55029009:C:T | -0.02448 | 0.01314 | -1.863 | 0.062865 | . |
| chr1:55062956:G:A | -0.0847 | 0.02311 | -3.665 | 0.000264 | *** |
| chr1:55064198:C:T | 0.08993 | 0.03698 | 2.432 | 0.015233 | * |
| chr1:55087895:T:C | 0.09855 | 0.04523 | 2.179 | 0.029636 | * |
|  |  |  |  |  |  |
| Estimates refer to age-adjusted log(PCSK9) residues | | | |  |  |

**Supplementary Table S2. Colocalization analysis with GTEX.**

| **Associated Loci** | **Tested genes** | **Best colocalization signal** | **Target tissue** | **P-value** |
| --- | --- | --- | --- | --- |
| chr1:206661203-207161203 | IL10, RASSF5, IL19, IKBKE, DYRK3, EIF2D, C1orf147, MAPKAPK2, IL20, IL24, FAIM3, PIGR, FCAMR, Y_RNA, RP11-343H5.4, RP11-564A8.4, RP11-534L20.4, RP11-343H5.6, SNORD112. RP11-534L20.5, RP11-564A8.8 | None | None | - |
| chr1:52247077-52747077 | NRD1, OSBPL9, TXNDC12, BTF3L4, ZFYVE9, RAB3B, KTI12, RNA%%P48, MIR761, Y_RNA, ANAPC10P1, RP11-91A18.4, RP4-800M22.2, PDCL3P6, TXNDC12-AS1, RP4-800M22.1, RP11-91A18.1, TSEN15P2, RP4-800M22.4, DNAJC19P7, RN7SL788P, RN7SL290P, AL589663.1, RP4-657D16.3, RP4-657D16.6 | None | None | - |
| chr1:95519375-96019375 | RWDD3, TMEM56, ALG14, Y_RNA, AL356479.1, RP11-57H12.3, RP4-586015.1, RP11-57H12.5, RP11-286B14.1, RP11-313A24.1, RP11-14019.1, RP11-14019.1, RP11-57H12.2, RP4-617C6.1, RP11-14019.2 | RWDD3 | Adrenal, Brain, skeletal muscle | 0,04 |
| chr7:105083050-105583050 | PUS7, CDHR3, RINT1, ATXN7L1, EFCAB10, RP11-251G23.2, CTA-351J1.1, YBX1P2, RP11-251G23.5 | ATXN7L1 | thyroid | 0,09 |
| chr9:113251372-113751372 | LPAR1, MUSK, RNU6-124P, RNU6-432P, RP11-410K21.2, RP13-461N9.2, SVEP1 | none | none | - |
| chr12:4858422-5358422 | KCNA1, GALNT8, KCNA5, KCNA6, RP3-377H17.2, RP11-429A20.2,RP11-319E16.1, RP11-429A20.4, RP11-429A20.3, RP11-234B24.4 | KCNA1 | adipose subcutaneous | 0,0006 |
| chr13:89938022-90438022 | LINC00353, LINC01040, RP11-75N6.3, SP3P | none | none | - |
| chr14:35380562-35880562 | PPP2R3C, SRP54, KIAA0391, PSMA6, NFKBIA, FAM177A1, IGBP1P1, RP11-561B11.1, RPL9P3, RP11-173E17.3, RP11-85K15.2, RP11-561B11.3, RP11-173D9.5, AL121594.1 | FAM177A1 | Fibroblasts | 0,0000004 |
| chr18:34540416-35040416 | CELF4, KIAA1328, RP11-797E24.3, RP11-9502.1, RP11-9502.5 | none | none | - |
